# Effects of Two Teaching Strategies on Preschoolers’ Oral Language Skills: Repeated Read-Aloud With Question and Answer Teaching Embedded and Repeated Read-Aloud With Executive Function Activities Embedded

**DOI:** 10.3389/fpsyg.2019.02932

**Published:** 2020-01-21

**Authors:** Hsin Ying Chien

**Affiliations:** Department of Early Childhood Education, National Taitung University, Taitung, Taiwan

**Keywords:** read-aloud, question and answer, executive function, oral comprehension, receptive vocabulary

## Abstract

The purpose of this study was to investigate the impact of two teaching strategies on preschoolers’ oral language skills: repeated read-aloud with question and answer teaching embedded, and repeated read-aloud with executive function (EF) activities embedded. A quasi-experimental pretest–posttest design was employed. Children ranging in age from 4 years 6 months to 6 years and 4 months participated in the study (*n* = 53). They were recruited from preschools in Taitung, Taiwan, randomly assigned to the three study groups. 36 children were divided and assigned to the two experimental groups (question and answer teaching group and EF group), while the remaining 17, to the control group. The participating preservice teachers attended 32 h of training which included the theory, practice, and outcome evaluation measure for the teaching strategies implemented. The intervention spanned 2 months. Oral language tests (curriculum-based receptive vocabulary, inferential comprehension, and oral comprehension) were administered before and after the intervention. The findings revealed that both experimental groups positively impacted participants’ receptive vocabulary and oral comprehension when compared with the control group, although the performances between the two experimental groups did not differ significantly. For inferential comprehension, there was no statistically significant difference across the three groups. Implications of the study findings are discussed and potential topics for future research proposed.

## Introduction

Reading books aloud to children by parents or teachers is one of the most recommended practices to help children learn languages (Snow et al., 1998; Whitehurst et al., 1999; Neuman et al., 2000). Research has shown that read-aloud can help increase children’s vocabulary (Beck et al., 2002; Biemiller and Boote, 2006; Pollard-Durodola et al., 2011) and oral comprehension skills (Cunningham and Stanovich, 1997; Morrow and Gambrell, 2002). A meta-analysis by the National Early Literacy Panel (2009) reported that reading aloud to children by teachers or parents can raise children’s verbal language abilities and knowledge acquired from print materials.

Reading picture books aloud provides children with a richer context to gain understanding of a word or text. However, during the initial round of read-aloud, children’s “encounter” with the word and text remains largely incidental, characterized by a partial and limited impression formed in children’s cognition of the pronunciation, semantics, and use of the new word and sentences strung together by these words. Only through repeated read-aloud can the process of encoding be reinforced and the process of acquiring new vocabulary and textual information become complete. These new words and textual information will be stored in children’s memory, and can be retrieved when children come across the same or similar contexts in the future (Sénéchal, 1997). Some scholars believe that a minimum of three rounds of read-aloud will allow children to internalize the meanings of the text or vocabulary (Hoffman, 1976).

Biemiller and Boote (2006) reviewed and analyzed 13 studies from 1989 to 2002, and found that repeated read-aloud combined with explaining the vocabulary explicitly resulted in an average increase of 26% in the learning of word meanings among primary graders. Similar observations were also made among students who listened to a text, received explanations of the target vocabulary in the context of the text, and made greater strides in learning the vocabulary (Nagy et al., 1987).

Beyond the learning of vocabulary, question and answer (Q&A) teaching where teachers ask questions and students answer is often implemented to deepen students’ text comprehension (Hansen and Pearson, 1983; van den Broek et al., 2001; McGee and Johnson, 2003; Richards and Anderson, 2003). Asking questions has long been used as a tool to not only promote comprehension but also assess students’ knowledge and stimulate critical thinking (Raphael and McKinney, 1983). Q&A both facilitates students’ comprehension of the content pertaining to the questions directly and enhances students’ comprehension and memory of the text as a whole (van den Broek et al., 2001). Teachers often engage listeners with different kinds of questioning, by targeting different levels of comprehension as espoused by Kintsch (1998) and formulating the questions accordingly.

Meanwhile, executive functions (EFs) have been shown to play an essential role in effective early academic instruction. Oral comprehension is a complex process which activates such cognitive functions as encoding, discriminating, flexibility (in switching between verbal, meaningful codes), and inference (Cain et al., 2004). EFs also help an individual to stay on task. EFs consist of a multitude of components, such as working memory, inhibitory control, and cognitive flexibility (Miyake et al., 2000). Some scholars believe that EFs are closely related to preschoolers’ vocabulary acquisition (Wolfe and Bell, 2004; Moriguchi et al., 2008) and can serve as a predictor of school children’s oral comprehension ability in the classroom (Hungerford et al., 2012). The dramatic growth in EFs between the ages of three and 5 years (Center on the Developing Child, 2012) has now been widely recognized. Scholars have also found that EF activities facilitated the early development of literacy among kindergarteners (Foy and Mann, 2013).

Executive functions can be enhanced through cognitive curricula (Barnett et al., 2008; Goldin et al., 2014). Research has also shown that domain-specific EF training targeting a particular EF component can yield the most change in learning behaviors (Wass, 2015). Blair and Razza (2007) studied a group of 3- to 5-year old children from low-income households to examine the role of self-regulation in their emerging academic abilities. They found that inhibitory control training benefited children’s learning of vocabulary. They went on to suggest that curricula designed to improve self-regulation as well as early literacy abilities might be more effective in enhancing children’s learning. This finding has been echoed in other studies (Segers et al., 2016). Empirical evidence has also been found that behavioral control training can significantly improve children’s performance in reasoning-related tests (Liu et al., 2015). Hence, one of the goals of the current study was to examine whether EF training could lead to improvement in more advanced language skills, such as oral comprehension and inferential comprehension, in addition to youngsters’ vocabulary acquisition.

There has been some empirical evidence with regard to the effects of Q&A teaching for language learning among school age children and preschoolers (Wang, 2012; Lee and Chien, 2019). On the other hand, despite the sound reasoning presented in the above literature review of the potential correlation between enhanced EFs and language learning, there remains little empirical evidence to confirm this association. Furthermore, any positive effect of robust EFs on language learning will be indirect, that is, through the mediation of enhanced cognitive functions. Hence, such benefits may take longer to manifest. Meanwhile, embedded instruction has been shown to benefit children’s learning and lead to greater academic achievement (McClelland et al., 2007; Raver et al., 2011). Therefore, our research hypotheses were formulated as follows:

(a) Within the study period, both the repeated read-aloud with Q&A embedded and repeated read-aloud with EF activities embedded more positively impact the curriculum-based vocabulary measure among children than repeated read-aloud alone; the repeated read-aloud with Q&A embedded also more positively impacts the vocabulary measure than the repeated read-aloud with EF activities embedded;
(b) Within the study period, both the repeated read-aloud with Q&A embedded and repeated read-aloud with EF activities embedded more positively impact the inferential comprehension measure among children than repeated read-aloud alone; the repeated read-aloud with Q&A embedded also more positively impacts the inferential comprehension measure than the repeated read-aloud with EF activities embedded; and
(c) Within the study period, both the repeated read-aloud with Q&A embedded and repeated read-aloud with EF activities embedded more positively impact the oral comprehension measure among children than repeated read-aloud alone; the repeated read-aloud with Q&A embedded also more positively impacts the oral comprehension measure than the repeated read-aloud with EF activities embedded.

## Methods

### Research Participants

#### Student Participants

Fifty-three participants were recruited from two preschools in Taitung County (Taiwan) for this study, aged from 4 years 6 months to 6 years and 4 months. Using a random number generator, participants were assigned to the Q&A group (Experimental Group I) (*n* = 18), the EF group (Experimental Group II) (*n* = 18), or the control group (*n* = 17). The average age was 65.58 months (*SD* = 8.06). Informed consent was obtained from all parents. Exclusion criteria included hearing impairment, other types of special needs, or serious behavioral problems. The Peabody Picture Vocabulary Test-Revised (PPVT-R) (*M* = 110.51, *SD* = 13.52) scores of student participants were within the normal range. As EFs and language comprehension could vary with age (Fuster, 2002; Berk, 2006), pre-intervention analysis was conducted and no statistically significant age differences were found across the study groups [*F*(2,52) = 0.12, *p* = 0.89]. Participants’ demographic information is presented in Table 1.

**TABLE 1:** Background information to group participants.

**Group**	**Gender**		**Age-month M (SD)**	**PPVT M (SD)**	**HTKS M (SD)**
	**Male**	**Female**			
	***n (%)***	***n (%)***			
Experimental I (*n* = 18)	10	8	66.22	109.39	0.64
(Question&Answer)	(56%)	(44%)	(9.35)	(13.64)	(0.33)
Experimental II (*n* = 18)	7	11	64.89	111.06	0.42
(Executive function)	(39%)	(61%)	(7.24)	(11.33)	(0.27)
Control (*n* = 17)	9	8	65.65	111.12	0.59
	(53%)	(47%)	(7.85)	(16.05)	(0.37)
Total	26	27	65.58	110.51	0.55
(*n* = 53)	(49%)	(51%)	(8.06)	(13.52)	(0.32)

*n, Number of participating children; M, mean score; SD, standard deviation; HTKS, Head-Toes-Knees-Shoulders.*

#### Instructors

A total of 10 instructors participated who were all senior-level preservice teachers at the university with which the author of this manuscript is affiliated. Instructors completed 32 h of training which covered three aspects—basics for pedagogical knowledge and pedagogical content knowledge, practice teaching and outcome measure—of the teaching strategies to be implemented: repeated read-aloud, repeated read-aloud with Q&A embedded, and repeated read-aloud with EF activities embedded (Table 2). All instructors attended the basics and outcome measure sessions, because they would be the test administrators of the study groups to which they were not assigned. Hence, they needed a general understanding of the different teaching strategies as well as the testing procedure and its relationship to the curriculum goal.

**TABLE 2:** Instructor training program.

**Session**	**Theme**	**Description**	**No. of hours**
Basics (all instructors)	Introduction 1: pedagogical knowledge	Language learning	2
		Relationship between read-aloud/repeated read-aloud and language learning	2
		Executive function: definition and components	2
		Relationship between executive function and language learning	2
	Introduction 2:pedagogical content knowledge of teaching strategies	Teaching strategy of repeated read-aloud: principle and lesson plan (using picture books)	2
		Teaching strategy of repeated read-aloud with Q&A embedded: principle and lesson plan (using picture books)	3
		Teaching strategy of repeated read-aloud with EF activities embedded: game design and lesson plan (using picture books)	3
Practice (group sessions)	Demonstration and hands-on practice teaching	Demonstration of the group-specific teaching strategy (by the researcher or the session leader)	3
		Practice among paired instructors (session videotaped)	3
		Teaching rehearsal (before the researcher) by each instructor based on the lesson plan specific to the picture books selected (session videotaped)	4
Evaluation (all instructors)	Outcome measure instruments	Review of the manuals of test instruments and demonstration (by the researcher staff)	6
		One-on-one practice (between the researcher and the instructor) of giving instructions when administering tests	
		Hands-on practice among paired instructors	
Total			32

Instructors stayed with their respective groups through the completion of the study. Implementation fidelity was ensured prior to and during the interventions. Instructors were supervised in a group setting every 2 weeks. Fidelity monitoring was also carried out through sporadic in-class observations and one-on-one supervision. The researcher provided additional intensive one-on-one guidance to instructors who did not have prior experience in administering related curricula. All teaching sessions were videotaped. The treatment fidelity among instructors was as high as 84%, which met the requirement for the program.

### Research Design

This quasi-experiment compared preschoolers’ performance in oral language and comprised three program groups: the Q&A group (Experimental Group I), the EF group (Experimental Group II), and control group.

Experimental group I: “repeated read-aloud,” “teaching vocabulary,” and “Q&A teaching”;

Experimental Group II: “repeated read-aloud,” “teaching vocabulary,” and “EF activities”;

Control Group: “repeated read-aloud” only.

### Interventions

The interventions spanned eight weeks, with four sessions per week. A total of 32 sessions were completed and each session lasted approximately 30 min. Each picture book was read to participants aloud in three rounds during the study. Respective interventions were carried out in the three study groups as follows.

#### First Round

##### Experimental groups I and II

The instructor explained and discussed new vocabulary by using picture cards as well as synonyms and antonyms to help students recognize the vocabulary. The picture book was then read aloud to students, followed by students playing the morpheme game.

##### Control group

The instructor read the picture book aloud, without additional interventions.

#### Second Round

##### Experimental group I

The instructor read the picture book aloud to participants, and then asked participants to answer a number of questions (for examples: what, who, what, etc.) which were formulated based on the text.

##### Experimental group II

The instructor read the picture book aloud. The instructor then asked participants to pay attention and memorize the meanings of the vocabulary discussed during the first stage, before engaging participants in a word game where participants were instructed to act out the correct response. For example, participants would clap hands when the instructor said the word “white” and stomp their feet when the instructor said the word “black.” This procedure was repeated three to five times until students mastered the one-to-one relationship between the body movement and the corresponding vocabulary. Afterward, participants responded with the body movement that was opposite what they learned earlier in this stage. For example, when the instructor said the word “black,” students would clap hands; when the instructor said the word “white,” participants would stomp their feet. Once participants mastered the rules, they played the game for six times using the synonyms and antonyms derived from the vocabulary in the picture book.

##### Control group

The instructor read the picture book aloud, without additional interventions.

#### Third Round

##### Experimental group I

The instructor read the picture book aloud to student participants, followed by inferential inquiries. The instructor guided the participants to distil hidden meanings in the text by asking questions like “why” and “how.” The instructor also encouraged students to identify causal relationships in the text by utilizing clues provided in the text as well as students’ existing background knowledge.

##### Experimental group II

The instructor read the picture book aloud to the participants. The instructor then mixed types of statements—active/passive and simple/complex—and presented children with four kinds of sentences: active plausible, active implausible, passive plausible, and passive implausible (Ye and Zhou, 2008). Children were asked to present their judgment of the veracity of the sentences by raising the signs of “correct” or “false.” This exercise also allowed the instructor to gauge participants’ grasp of the storyline in the picture book as a whole. There was a total of five such exercises.

##### Control group

The instructor read the picture book aloud, without additional interventions.

### Instruments

A battery of tests were used to examine student participants’ oral language abilities and EF abilities. All tests have been standardized and validated. PPVT-R and The Head-Toes-Knees-Shoulders test were administered during the two weeks prior to the start of interventions (“pretest”), due to the scheduling. The remaining tests were administered during both the pretest and post-test which also lasted two weeks immediately after the completion of interventions, due to the scheduling.

#### Peabody Picture Vocabulary Test-Revised (PPVT-R)

The screening instrument used to recruit participants was a Mandarin Chinese version of the PPVT (Dunn and Dunn, 1981), which was standardized and revised by Lu and Liu (1994). The test is suitable for measuring the receptive vocabulary comprehension of children aged 3 to 12, by having them point to one of the four picture cards which best represents the word spoken to them. The reliability of the PPVT-R as reported in the manual is 0.88. The test took children in this study 15 to 22 min to complete.

#### Executive Function Measure

The Head-Toes-Knees-Shoulders (HTKS) task was conceptualized by Ponitz et al. (2008) as a measure of EFs, including inhibitory control (a child must inhibit the dominant response of imitating the examiner), working memory (a child must remember the rules of the task), and attention focusing (a child must pay attention to the directions being presented by the examiner). First, one of the participating instructors (a senior in college who was knowledgeable about the HTKS task) engaged with children in groups (of 17–18 children each) to practice the HTKS:

Practice 1: The instructor directed children to touch their head (or their toes), but instead of following the command, the children were supposed to do the opposite and touch their toes. This round was repeated three times.
Practice 2: Once participants mastered the head/toes part of the task, the knees and shoulders commands were presented to them. This round was repeated three times.
Practice 3: Once children mastered the knee/shoulder part of the task, all commands were combined. Once again, children were supposed to do the opposite of what the instructor said. This round was repeated four times.

After the practice sessions, 3–5 participating instructors (different from the instructor who led the practice sessions) administered the test by conducting it with each child one on one at a quiet corner in the classroom. The HTKS task was carried out 10 items during the test session and scored as follows: two points for responding correctly the first time; one point for responding incorrectly the first time, but enacting rectification right away; and zero point for responding incorrectly, without rectification. The internal consistency reliability (Cronbach’s α) was 0.95.

#### Oral Language Measures

##### Curriculum-based picture vocabulary test

This instrument was created by the research team to assess participants’ vocabulary comprehension. This computer-assisted test was designed using the Java programing language and administered individually. The questions (50) were modeled after those in the PPVT, and participants score by clicking on the correct picture after listening to the test questions. Five questions were created for each picture book. Each question contains three choices and counts for one point. The total score is indicative of participants’ performance in vocabulary comprehension. This test took participating children 6.5 to 12 min to complete in this study. A Shapiro–Wilk test showed *W*(53) = 0.98, *p* = 0.44 (greater than 0.05), indicating the normality of data. The points on the Q–Q plot graph closely follow the normal distribution line (Gravetter and Wallnau, 2014).

##### Oral comprehension test for young children

This instrument (Chien et al., 2017) was designed to test oral comprehension among children aged 4–6 years. It has been standardized for the Mandarin Chinese-speaking children in Taiwan (*n* = 352). Children first listened to the instructions and answered practice questions. Once they became familiar with the process and made no errors, the official test began.

The test includes three short texts and five dialogic texts with equal difficulty. The texts feature three themes: food, kindergarten activity, and holiday. The short text and dialogic text each contain 144–170 words, and were read to participants using an audio recorder. For each text read, two to four questions were posed to participants who then located answers in the text (by either identifying a specific word/phrase or stringing together clues scattered in the text). For each question, there are three answers for children to choose from. Children pointed to the answer of their choice on the answer sheet and the test administrator recorded the answer. The test took participants 20 to 30 min to complete in this study.

The discrimination indices range from 0.27 to 0.51, and the difficulty indices, from 0.29 to 0.82. The internal consistency reliability (Cronbach’s α) is 0.81 and the test–retest reliability coefficient is 0.68. Both are acceptable reliability measurements. The test items also fit the Rasch model. A Shapiro–Wilk test showed *W*(53) = 0.97, *p* = 0.23 (greater than 0.05), indicating the normality of data. The points on the Q–Q plot graph also closely follow the normal distribution line.

##### Inferential comprehension test for young children

This instrument was developed based on the inferential comprehension test for young children created by Chien et al. (2017) for kindergarteners aged 4 to 6, by also taking into consideration the recommendations of Florit et al. (2011) as well as Silva and Cain (2015). It has been standardized for the Mandarin Chinese-speaking children in Taiwan (*n* = 234). Participants first listened to directions and went through a practice session. Once no more errors were made, the formal test began.

The test comprises short sentences which are read to participants using an audio-recorder. For example, “Xiaoming took a glass of milk and accidentally kicked the chair,” “What happened, please?,” “Mom brought a rag,” and “What does Mom do with a rag?” Test questions were posed to participants after each sentence. There are 20 questions for the pretest, and 22 questions for the post-test. To answer the questions, participants needed to identify explicit clues in the sentences (text-based comprehension) and/or retrieve relevant information from the existing pool of their background knowledge (knowledge-based comprehension). The test administrator then recorded participants’ answers. After the test, the administrator scored the answer sheet using a two-point scale (0 or 1). The sum of the scores indicates participants’ comprehension performance. The higher the sum, the stronger the inferential comprehension. The test took participants 25 to 33 min to complete in this study.

The discrimination indices range from 0.30 to 0.56 and the internal consistency reliability (Cronbach’s α) is 0.82. Pearson’s coefficient of skewness of 0.18 and the coefficient of kurtosis of 0.05 indicate a normal univariate distribution. A Shapiro–Wilk test showed *W*(53) = 0.97, *p* = 0.15 (greater than 0.05), indicating the normality of data. The points on the Q–Q plot graph also closely follow the normal distribution line. There are two equivalent sets of test form which could be used for testing before and after the intervention.

### Materials

The materials used for read-aloud were mostly picture books containing scientific information or content close to children’s daily life. Initially, 28 titles were recommended by three senior kindergarten teachers. Ten of them were later selected by two experts in the fields of early childhood language intervention and natural science, respectively (Lee and Chien, 2019). Seven of the 10 picture books were read aloud in the following order: (1) “Rosie’s Walk” (Hutchins, 2009), (2) “The Soles of Your Feet” (Yagyu, 1987a), (3) “Your Hand and Fingers” (Yagyu, 1987a, b), (4) “The Story of Bones” (Horiuchi, 2013), (5) “Miki’s First Errand” (Hayashi, 2010), (6) “Where is the Rhinoceros Beetle?” (Matsuoka, 2011), (7) “The Story of Fart”” (Cho, 1984) (Appendix). The remaining three picture books used were compiled by researchers of this study based on the information extracted from encyclopedias or professional natural science websites. The titles of these three books were “Burp,” “Grow Hair,” and “Microwave,” and read aloud to children in this order. The content was verified by two experts in applied science and healthcare, respectively, to ensure the accuracy of texts and illustrations.

The text length ranges from 383 to 418 words, and the sentence count, from 31 to 37. There are on average 18.9 difficult words in the text, equivalent to the reading level of second- to third-graders (in general, children’s listening comprehension level is higher than their level of reading comprehension). The vocabulary chosen from the books for further discussion were deemed important to understanding the story in the book (Kuhn and Stahl, 1998).

### Data Analysis

Analysis of covariance (ANCOVA) was conducted using the experimental groups (2) and control group as independent variables, pretest scores as covariates, and scores of the outcome measures as dependent variables.

## Results

The descriptive statistics for all measures and study groups before and after the interventions are tabulated in Table 3. The pretest scores were used as covariates in the data analysis.

**TABLE 3:** Mean scores (SDs) at pre-test, post-test, and adjusted means at post-tests for orallanguage skills.

	**Pre-test**			**Post-test**			***F***	***p***	***η^2^***
**Test items (number of questions)**	**Experiment I (*n*** = **18)**	**Experiment II (*n*** = **18)**	**Control (*n*** = **17)**	**Experiment I (*n*** = **18)**	**Experiment II (*n*** = **18)**	**Control (*n*** = **17)**			
1-1 CB^1^ Receptive vocabulary (50)	23.61 (4.06)	23.53 (4.89)	24.61 (4.45)	34.83 (3.91)	30.47 (5.39)	28.94 (5.99)	3.41^∗^	0.04	0.129
				34.88^a^	30.67^a^	28.25^a^			
1-2 Inferential comprehension (39)	23.39 (4.90)	21.65 (5.74)	20.66 (6.65)	32.56 (4.09)	27.94 (4.37)	24.72 (5.59)	0.33	0.72	0.014
				31.67^a^	28.07^a^	25.53^a^			
1-3 Oral comprehension (25)	13.67 (2.57)	13.29 (1.83)	14.17 (2.57)	21.78 (3.15)	21.71 (2.64)	16.67 (3.69)	6.17^∗∗^	0.004	0.212
				21.83^a^	22.17^a^	15.06^a^			

*CB^1^, curriculum-based; ^*a*^adjusted means of post-tests;^∗^*p*-value <0.05; ^∗∗^*p*-value <0.01.*

There were no significant differences in all pretest measures across the study groups: curriculum-based picture vocabulary [*F*(2,47) = 3.20, *p* > 0.05], oral comprehension [*F*(2,47) = 1.06, *p* > 0.05], and inferential comprehension [*F*(2,47) = 0.233, *p* > 0.05]. The homogeneity of regression slopes assumption was also tested and met. The interactions between the covariate and the groups for each measure (dependent variable) were as follows: *F*(2,47) = 0.76, *p* = 0.47, η^2^ = 0.26 (curriculum-based picture vocabulary), *F*(2,47) = 1.08, *p* = 0.20, η^2^ = 0.08 (oral comprehension), and *F*(2,47) = 0.62, *p* = 0.54, η^2^ = 0.03 (inferential comprehension). Where pre- and post-test scores differed significantly, the effect sizes fell between small and medium ranging from 0.128 to 0.212.

### Impact of the Interventions on Receptive Vocabulary

The results of ANCOVA revealed a significant main effect [*F*(2,46) = 3.41, *p* = 0.04, η^2^ = 0.129, observed power = 0.72]. *Post hoc* analysis further revealed that post-test scores of both the Q&A group (34.83) and the EF group (30.47) were significantly higher than the score of the control group (28.94). However, there was no significant difference between the two experimental groups.

### Impact of the Interventions on Inferential Comprehension

The difference across the three study groups did not reach statistical significance [*F*(2,46) = 0.33, *p* = 0.72], indicating no significant contributory effect of either intervention. Hence, no further analysis was conducted.

### Impact of the Interventions on Oral Comprehension

Analysis results showed a main effect [*F*(2,46) = 6.17, *p* = 0.004, η^2^ = 0.212, observed power = 0.89], indicating significant contributory effects of different teaching strategies on children’s oral comprehension. Based on *post hoc* analysis, the post-test scores in the Q&A group (21.83) and the EF group (22.17) were significantly higher than the score in the control group (15.06). However, there was no significant difference between the two experimental groups.

## Discussion and Implications

The purpose of this study was to examine the effects on various oral language abilities of two interventions: repeated read-aloud with Q&A embedded, and repeated read-aloud with EF activities embedded. The study found that the experimental interventions improved participants’ curriculum-based receptive vocabulary and oral comprehension.

The higher post-test scores in receptive vocabulary in both the Q&A and EF groups (employing both read-aloud and vocabulary teaching) than the score in the control group (employing only read-aloud) confirmed the findings from earlier studies that instructors need to, in addition to reading aloud, explain the vocabulary and its contextual meaning in the text in order to effectively promote children’s vocabulary acquisition (Hargrave and Sénéchal, 2000; Dickinson and Tabors, 2001; Penno et al., 2002; Biemiller and Boote, 2006; Lee and Chien, 2019). This study is evidence that picture cards, morpheme games can be an effective strategy to explain vocabulary in the preschool age group. Instructors in both experimental groups in this study used picture cards, synonyms, antonyms, and morpheme games during the first stage of intervention to illustrate in greater depth the meanings of the vocabulary. These meanings were then reinforced through the WH questions in the Experimental Group I (Q&A) and EF play in the Experimental Group II during the second stage of the intervention, respectively.

The oral comprehension scores among participants in the two experimental groups improved significantly after the interventions, which also echoes the findings from earlier research (Morrow and Gambrell, 2002).

However, the scores in inferential comprehension in the three study groups did not differ significantly. Inferential comprehension requires readers or listeners to draw from their existing background knowledge which pertains to the topics in the text (Carr and Thompson, 1996). Comprehending science-related texts (as in some of the materials used in this study) relies even more on making inferences from existing background knowledge. Instructors shall also be instrumental in guiding children to generate knowledge-based or text-based inferences by encouraging children to connect information presented in non-adjacent parts in the text (McNamara and Magliano, 2009; Bos et al., 2016). In the current study, the requirement to adhere to the teaching protocol might have hindered instructors from engaging in more robust exchange with children to assist children in tapping into their existing knowledge pool and making inferences. The lack of statistically significant difference across the study groups in children’s inferential comprehension performance could also be linked to the absence of predictive discussion prior to the read-aloud (Hansen and Pearson, 1983). When combined with Q&A after the read-aloud, predictive discussion has been shown to help participants more effectively connect their background knowledge with the text read to them, so a cohesive and meaningful mental representation of the text can be created to deepen the comprehension.

Interestingly, no significant difference in the vocabulary learning and oral comprehension was found between the two experimental groups. As any potential benefit to language learning from EF activities will be indirect through the mediation of important cognitive functions (as presented in the introduction section of this report), such benefit may take longer to manifest than the time frame selected for the current study. Another plausible contributor to this lack of difference might lie in the small sample size used for this study. Hence, follow-on research can track a larger group of participants over a longer time horizon to further confirm or challenge our research hypotheses.

We believe that the present study contributes to the existing body of literature by providing empirical evidence that teaching strategies of repeated read-aloud with Q&A teaching or EF activities embedded can improve preschoolers’ oral language abilities (in particular, vocabulary and oral comprehension). This finding once again highlights the complexity in instructor-student interactions encompassing linguistic and cognitive processes (non-linguistic), and both are required for children to successfully acquire oral language skills. The teaching strategies implemented in the Q&A group embody the linguistic component—that is, utilizing linguistic resources such as pictures, synonyms, and antonyms to facilitate and deepen children’s learning during the early literacy phase. On the other hand, the evidence emerging from the current study also supports the benefits delivered by enhanced EF faculties in acquiring oral language skills. This mirrors findings from earlier research that EF activities focusing on a particular component of EF (for example, inhibitory control, as in this study) can be incorporated into teaching to enhance children’s learning of vocabulary. Children’s inhibitory control can contribute positively to their mastery of the more complicated learning, for example, a curriculum that features the learning of combinations of simple/complicated and active/passive sentences (as seen in the Experimental Group II in this study). Therefore, the preliminary findings of the current study could potentially further expand teachers’ toolbox by arming them with more teaching ammunitions: the strategies of reading-aloud and Q&A which are more commonly utilized in today’s classrooms (albeit not in combination), and the more innovative approach by incorporating EF games into the teaching of oral language skills.

Meanwhile, there are a number of limitations in the present study. First, the effect sizes ranged from small to medium where significant differences in pre- and post-test scores were found, likely due to the small sample size. Thus, the preliminary findings in the current research will need to be confirmed in a larger study. Second, for the measure of oral language comprehension, a more open-ended approach based on free recall can be considered in follow-on studies, in addition to or instead of using pre-formulated tests as in this study, to gain a fuller picture of children’s curriculum-based text comprehension and oral comprehension. Third, future studies could recruit in-service teachers who would likely be better prepared to manage young children’s responses and understand their intent so as to enhance the classroom interactions and deepen the teaching effects. Fourth, to confirm the lasting effects of interventions, a follow-up after a longer interval could be initiated with study participants. In the current study, the post-test measures were conducted immediately after the completion of the interventions.

## Conclusion

Teaching strategies of repeated read-aloud embedded with Q&A or EF activities can benefit young children’s receptive vocabulary learning and oral comprehension.

## Data Availability Statement

All datasets generated for this study are included in the article/supplementary material.

## Ethics Statement

The studies involving human participants were reviewed and approved by the National Chen Kung University Human Research Ethics Committee, by project title “The effects of repetition, read-aloud, and executive function exercises in read-aloud on receptive vocabularies and listening comprehension for young children” (Approval No: NCKU HREC-F-105-032-2). Written informed consent to participate in this study was provided by the participants’ legal guardian/next of kin.

## Author Contributions

HC conceived and designed the study, organized the data, performed the statistical analysis, and wrote the manuscript.

## Conflict of Interest

The authors declare that the research was conducted in the absence of any commercial or financial relationships that could be construed as a potential conflict of interest.

## Appendix

### Picture Books Used in the Study

(1) Hutchins, Pat (2009). *Rosie’s Walk* (Hsinex International Corporation, Trans.).
Taipei City, Taiwan: Hsinex International Corporation.
(2) Matsuoka, Tatsyhide (2011). *Where is the Rhinoceros Beetle?* (Y. W. Huang, Trans.).
Taipei City, Taiwan: Children’s Publications, Co., Ltd.
(3) Horiuchi, Seiichi (2013). *The Story of Bones* (Han Sheng Publisher, Trans.).
Taipei City, Taiwan: Han Sheng Publisher.
(4) Hayashi, Akiko (2010). *Miki’s First Errand* (M. Z. Lin, Trans.).
Taipei City, Taiwan: Han Sheng Publisher.
(5) Yagyu, Genichiro (1987). *The Soles of Your Feet* (Han Sheng Publisher, Trans.).
Taipei City, Taiwan: Han Sheng Publisher.
(6) Cho, Shinta (1984). *The Story of Fart* (Han Sheng Publisher, Trans.).
Taipei City, Taiwan: Eco Publishing, Co., Ltd.
(7) Yagyu, Genichiro (1987). *Your Hand and Fingers* (Han Sheng Publisher, Trans.).
Taipei City, Taiwan: Eco Publishing, Co., Ltd.
