# Corrigendum: Effects of Two Teaching Strategies on Preschoolers' Oral Language Skills: Repeated Read-Aloud With Question and Answer Teaching Embedded and Repeated Read-Aloud With Executive Function Activities Embedded

**DOI:** 10.3389/fpsyg.2020.00804

**Published:** 2020-04-28

**Authors:** Hsin Ying Chien

**Affiliations:** Department of Early Childhood Education, National Taitung University, Taitung, Taiwan

**Keywords:** read-aloud, question and answer, executive function, oral comprehension, receptive vocabulary

In the original article (Lee and Chien, 2019) was not cited. The citation has now been inserted in the paragraphs below:

The **Methods** section, subsection **Materials**, paragraph 1:

“Initially, 28 titles were recommended by three senior kindergarten teachers. Ten of them were later selected by two experts in the fields of early childhood language intervention and natural science, respectively (Lee and Chien, 2019).”

The **Discussion and Implications** section, paragraph 2:

“The higher post-test scores in receptive vocabulary in both the Q&A and EF groups (employing both read-aloud and vocabulary teaching) than the score in the control group (employing only read-aloud) confirmed the findings from earlier studies that instructors need to, in addition to reading aloud, explain the vocabulary and its contextual meaning in the text in order to effectively promote children's vocabulary acquisition (Hargrave and Sénéchal, 2000; Dickinson, 2001; Penno et al., 2002; Biemiller and Boote, 2006; Lee and Chien, 2019). This study is evidence that picture cards, morpheme games can be an effective strategy to explain vocabulary in the preschool age group. Instructors in both experimental groups in this study used picture cards, synonyms, antonyms, and morpheme games during the first stage of intervention to illustrate in greater depth the meanings of the vocabulary. These meanings were then reinforced through the WH questions in the Experimental Group I (Q&A) and EF play in the Experimental Group II during the second stage of the intervention, respectively.”

The following paragraphs have been revised slightly due to the addition of the reference above.

The **Introduction**, paragraphs 7 and 8:

“Executive functions can be enhanced through cognitive curricula (Barnett et al., 2008; Goldin et al., 2014). Research has also shown that domain-specific EF training targeting a particular EF component can yield the most change in learning behaviors (Wass, 2015). Blair and Razza (2007) studied a group of 3- to 5-year old children from low-income households to examine the role of self-regulation in their emerging academic abilities. They found that inhibitory control training benefited children's learning of vocabulary. They went on to suggest that curricula designed to improve self-regulation as well as early literacy abilities might be more effective in enhancing children's learning. This finding has been echoed in other studies (Segers et al., 2016). Empirical evidence has also been found that behavioral control training can significantly improve children's performance in reasoning-related tests (Liu et al., 2015). Hence, one of the goals of the current study was to examine whether EF training could lead to improvement in more advanced language skills, such as oral comprehension and inferential comprehension, in addition to youngsters' vocabulary acquisition.

There has been some empirical evidence with regard to the effects of Q&A teaching for language learning among school-age children and preschoolers (Wang, 2012; Lee and Chien, 2019). On the other hand, despite the sound reasoning presented in the above literature review of the potential correlation between enhanced EFs and language learning, there remains little empirical evidence to confirm this association. Furthermore, any positive effect of robust EFs on language learning will be indirect, that is, through the mediation of enhanced cognitive functions. Hence, such benefits may take longer to manifest. Meanwhile, embedded instruction has been shown to benefit children's learning and lead to greater academic achievement (McClelland et al., 2007; Raver et al., 2011). Therefore, our research hypotheses were formulated as follows:”

Paragraph 5 of the **Introduction** has now been removed. It read:

“However, there has been no empirical evidence presented in the literature with regard to the effects of Q&A teaching for the learning of vocabulary or English alphabet. Similar research on the learning of Mandarin Chinese has also been focusing on school-age children (Wang, 2012). Given the prevalence of Q&A teaching in preschools and kindergartens as part of the early literacy program, one of the objectives of the current study was to examine, through experimental teaching research, the effects of Q&A teaching on vocabulary learning among preschoolers. The study also aimed to investigate, if children's comprehension is enhanced through such teaching, whether that enhancement is manifested beyond the surface level of a text and to a level which requires children to make inferences by drawing clues from the text read to them as well as tapping into their existing background knowledge.”

The author apologizes for this error and state that this does not change the scientific conclusions of the article in any way. The original article has been updated.
